# Interactions between Two Parasitoids of Tephritidae: *Diachasmimorpha longicaudata* (Ashmead) and *Psyttalia cosyrae* (Wilkinson) (Hymenoptera: Braconidae), under Laboratory Conditions

**DOI:** 10.3390/insects11100671

**Published:** 2020-10-02

**Authors:** Shepard Ndlela, Samira Abuelgasim Mohamed, Abdelmutalab G.A. Azrag, Paul Nduati Ndegwa, George Otieno Ong’amo, Sunday Ekesi

**Affiliations:** 1 International Centre of Insect Physiology and Ecology (*icipe*), P O Box 30772, Nairobi 00100, Kenya; sfaris@icipe.org (S.A.M.); agesmalla@icipe.org or sekesi@icipe.org (S.E.); 2 Department of Crop Protection, Faculty of Agricultural Sciences, University of Gezira, P.O. Box 20, Wad Medani 21111, Sudan; 3 School of Biological Sciences, College of Physical and Biological Sciences (Chiromo Campus), University of Nairobi, P. O. Box 30197, Nairobi 00100, Kenya; pndegwa@uonbi.ac.ke (P.N.N.); gongamo@uonbi.ac.ke (G.O.O.)

**Keywords:** interaction, *Bactrocera dorsalis*, interspecific competition, extrinsic competition, parasitism, competition

## Abstract

**Simple Summary:**

Wasps, generally referred to as “farmers friends” because of their ability to control devastating fruit flies, were imported from Hawaii and released in Kenya for the management of the invasive Oriental fruit fly *Bactrocera dorsalis*. It was noted that the introduced wasp had developed a liking for the native fruit fly species *Ceratitis cosyra* which already has a native wasp controlling it. We therefore undertook a study to understand the interaction of the introduced and native wasp on the invader Oriental and native fruit flies. Our results show that when the native wasp attacks the invader fruit fly, the wasp is killed because of the strong immune system of the oriental fruit fly. The good news is that the introduced wasp efficiently controls both the invader and the native fruit flies. We have discussed these findings based on various scientific principles which favor the co-existence of the two wasps without any detrimental effect on their survival. We have also suggested that more studies be conducted in open field conditions where both fruit flies occur in abundance and choices regarding which fruit fly species to attack are much wider.

**Abstract:**

The braconid wasp, *Diachasmimorpha longicaudata* (Ashmead), was introduced in Kenya from Hawaii for classical biological control of the invasive tephritid, *Bactrocera dorsalis* Hendel. Following reports that *D. longicaudata* had formed new associations with *Ceratitis cosyra*, laboratory experiments were conducted to assess the interaction between the introduced and the native parasitoid of *C. cosyra*; *Psyttalia cosyrae* (Wilkinson) under three scenarios: *B. dorsalis* only, *C. cosyra* only and mixed populations of the two species. Parasitoids were introduced to the host as sole, sequential and simultaneous releases. Host searching and probing events were five times higher for *D. longicaudata* than *P. cosyrae* with both hosts. Total parasitism was highest (78%) when *D. longicaudata* was released alone on *C. cosyra*, compared to 20% for *P. cosyrae* released on the same host. Releases of *P. cosyrae* on *B. dorsalis* resulted in 0% parasitism, compared to 64% parasitism by *D. longicaudata*. Specific parasitism for *P. cosyrae* was three times higher when *P. cosyrae* was released first in sequential releases on *C. cosyra* compared to when it was released after *D. longicaudata*. These findings suggest that the two parasitoids can both suppress *C. cosyra* but *B. dorsalis* acts as a reproductive sink for *P. cosyrae*. Our findings should form the basis of field investigations where options are much wider for both parasitoids.

## 1. Introduction

The Tephritidae have a worldwide distribution and comprise approximately 4000–4500 described species in 500 genera [1,2,3]. Of these, about 1500 species are known to be associated with fruits, but only 17% are of economic importance [4] causing severe direct and indirect damage resulting in yield reduction and loss of markets through quarantine restrictions imposed by importing countries [5,6,7,8,9,10].

In response to enormous losses incurred in the horticultural agro-industry, programs aimed at managing fruit damaging Tephritidae have been implemented worldwide, albeit with variable success. Some of the strategies that have been used widely include chemical control [11,12], orchard sanitation [13,14], baiting techniques [15,16,17,18], sterile insect technique [19,20,21,22], male annihilation technique [18,23,24,25], entomopathogenic fungi [26,27,28] and use of parasitoids [29,30,31] among others.

The need for cost effective, environmentally friendly and sustainable approaches to fruit fly management has greatly pushed pest management in the direction of strategies fitting this category or creating new prospects for tactical combinations in an integrated pest management (IPM) approach [32]. One such strategy is the use of parasitoids. A number of natural enemies are responsible for regulating pest populations in nature and have since been harnessed in programs involving captive mass rearing and intentional release for the purpose of controlling target insect populations. Parasitic wasps in the order Hymenoptera, family Braconidae have been used in the management of fruit flies of economic importance such as *Ceratitis* [30], *Anastrepha* [29] and *Bactrocera* [31] among others. The family is one of the largest in the Hymenoptera, comprising more than 15,000 described species with several other new species still being described [33,34]. Within the Braconidae is the subfamily Opiinae, a diverse group of koinobiont endoparasitoids of various cyclorrhaphous Diptera constituting about 1500 to 1900 species worldwide [35,36,37]. Out of this number, more than 100 species have successfully been reared on fruit-infesting Tephritidae [38] and several have been effective in controlling most fruit flies of economic importance [31,39,40,41,42,43,44,45,46,47]. Opiinae are the most preferred natural agents for fruit fly suppression because of their host specificity and high parasitism rates [48,49,50].

Two tephritid fruit fly species of economic importance, namely *Bactrocera dorsalis* (Hendel) and *Ceratitis cosyra* (Walker), occur in Kenya where they cause a combined fruit loss of up to 80% on mango depending on season, locality and mango variety [5]. To curb their menace, an IPM package targeting various stages of fruit fly development is being promoted and implemented in different mango growing areas, and consists of spot application of bait spray, male annihilation technique (MAT), biopesticide applications, orchard sanitation and parasitoid releases [25,51,52,53]. New components continue to be added to the package through active research and enhancement of the existing tactics. Of this package, farmers are encouraged to use at least three compatible components as single bullet approaches are seldom effective.

Two parasitoid species, namely the indigenous *Psyttalia cosyrae* (Wilkinson) and the exotic *Diachasmimorpha longicaudata* (Ashmead) can play a significant role in managing *C. cosyra* and *B. dorsalis,* respectively, in the Kenyan mango agro-ecosystem. *Psyttalia cosyrae* and *D. longicaudata* are both synovigenic, koinobiont larval-pupal parasitoids of *C. cosyra* and *B. dorsalis,* respectively [54]. The former parasitoid is a co-evolved natural enemy of *C. cosyra* (but parasitizes other fruit fly hosts) while *D. longicaudata* parasitises several fruit flies [29,30,55]. *Diachasmimorpha longicaudata* was introduced in 2009 in Kenya from Hawaii for the classical biological control of *B. dorsalis* but has also been reported to have formed a new association with the indigenous *C. cosyra* [31]. This situation provides additive effects to the management of *C. cosyra* but may provide challenges in the form of negative interactions between the indigenous parasitoid *P. cosyrae* and the introduced one *D. longicaudata. Diachasmimorpha longicaudata* is the most widely used control agent in biological control programs of Tephritidae in North and South America as well as their associated islands [55]. It was successfully introduced into the Americas and soon became one of the most important parasitoids in the control of *Anastrepha* species and *C. capitata* [56,57,58]. Once released and established, *D. longicaudata* populations become self-perpetuating and persist in the system for many generations. According to Oroño and Ovruski [59], the parasitoid was recovered in the north western region of Argentina 40 years after its initial release. Currently in Kenya, the parasitoid has been recovered on *B. dorsalis* as well as *Cosyra cosyra* (Walker).

Exotic parasitoids are often introduced into new agro-ecological systems with the aim of suppressing a target pest [60]. However, they sometimes form new associations with other hosts that are usually controlled by indigenous parasitoids. In nature, a single host species is sometimes attacked by parasitoids belonging to different genera or species, resulting in interactions whose outcomes are multifaceted [61,62]. For example, one or both species might seek to exclusively use host resources [63] thereby resulting in fierce competition at the level of seeking the host or among developing immature stages within the host [64].

In instances where the introduced and indigenous parasitoids share the same host, competition may either affect the establishment of the former or performance of the latter, resulting in the decline of reproduction and ultimate population drop of either of the two or even both depending on the severity of the contest [63,65,66]. In fact, a host attacked by a koinobiont parasitoid remains available to further parasitization by other generalist parasites of different species resulting in a contest for resources [67]. This phenomenon is of importance in determining host–parasitoid interactions that lead to the ultimate success of the natural enemy [67]. The implications of such associations are usually not fully explored, resulting in mysterious extinctions of native parasitoids at particular trophic levels. In the context of Kenya, indigenous parasitoids were unable to suppress *B. dorsalis*, due to encapsulation by the host [68,69], necessitating the importation and release of an exotic parasitoid to control *B. dorsalis*. The introduced parasitoid showed promising results in controlling the target pest and was able to successfully parasitize and complete its life cycle in the indigenous *C. cosyra*. Therefore, the objective of this study was to evaluate the interaction between the introduced parasitoid *D. longicaudata* and the indigenous *P. cosyrae* at various parasitoid–host combinations involving *B. dorsalis* and *C. cosyra*.

## 2. Materials and Methods

### 2.1. Hosts and Parasitoids

The fruit flies *B. dorsalis* and *C. cosyra*, as well as parasitoids *D. longicaudata* and *P. cosyrae* used in this study were reared at the International Centre of Insect Physiology and Ecology (*icipe*) mass rearing quarantine facility in Nairobi, Kenya. The flies were reared following the procedures described by Mohamed et al. [54], Ekesi et al. [70], Ekesi and Mohamed [71]. They were kept in Perspex cages (80 × 80 × 80 cm) and maintained at 26–28 °C, 60–70% RH and a photoperiod of L12: D12. Adult flies were fed on a mixture of artificial diet consisting of ground sugar and enzymatic yeast hydrolysate ultrapure (USB Corporation, Cleveland, OH, USA) in the ratio 3:1 by volume and were provided with water in a petri dish (8.6 cm in diameter) with a layer of pumice granules. Flies from wild populations were periodically added to the mass reared stock flies at 3-month intervals to maintain genetic variability. *Diachasmimorpha longicaudata* was reared on late second instar larvae of *B. dorsalis* while *P. cosyrae* was raised on *C. cosyra* larvae of the same age following a procedure similar to that described by Mohamed et al. [54] and Ekesi et al. [72]. Parasitoids were maintained at 25–27 °C, 60–70% RH and a photoperiod of L12: D12 in Perspex cages (40 × 40 × 40 cm) and provided with fine drops of pure honey streaked on the topside of the cages and water on moist cotton wool balls (5–6 cm in diameter).

### 2.2. Experimental Procedure

The interaction of the two parasitoids was determined under three scenarios (Table 1) of *B. dorsalis* only, *C. cosyra* only and mixed infestation of *B. dorsalis* and *C. cosyra* at a ratio of 1:1. Seven to nine and 13–15-day-old mated naive females of *D. longicaudata* and *P. cosyrae,* respectively, were used in all cases outlined below. The choice of the ages is based on experimental evidence and our experiences in mass rearing these two parasitoids. For *B. dorsalis* as host, five different set ups were evaluated as follows: (a) 100 late 2nd instar larvae of *B. dorsalis* were placed in an oviposition unit consisting of a customized petri dish (9 cm diameter and 0.3 cm depth) with a tightly fitting organza lid. The larvae were provided with a diet containing carrot powder (24.2 g), sugar (16.2 g), brewer’s yeast (8.1 g), citric acid (0.6 g), methyl p-hydroxbenzoate (0.2 g), and water (50.7 mL) [31,54]. Oviposition units were placed in ventilated Perspex cages (12 × 12 × 12 cm) and 20 females of *D. longicaudata* were released inside to forage and oviposit for 6 hr. (b) Secondly, 20 female parasitoids of *P. cosyrae* were released as in (a) above. (c) In the third set up, 10 females of *D. longicaudata* and another 10 of *P. cosyrae* were released simultaneously and allowed to oviposit. Furthermore, in the fourth set up (sequential release), (d) ten females of *D. longicaudata* were released first and allowed to oviposit for 3 hr, after which they were removed and the same number of *P. cosyrae* were also released and allowed to oviposit for the same period of time. (e) The fifth scenario was the same as scenario (d), except that *P. cosyrae* was released first (Table 1). In all cases, the control consisted of host larvae in which no parasitoids were introduced to determine natural host mortality as well as emergence rates. The experiments were replicated 11 times and procedures (a)–(e) repeated on *C. cosyra* as host as well as on combination of *B. dorsalis* and *C. cosyra* (Table 1). The number of parasitoids, landing, searching and ovipositing were recorded at 30-min intervals for 3hr, only in scenarios where the two parasitoids were not mixed. Each observation lasted 5 s. After the exposure period of 6 hr, the contents of the oviposition units were transferred to fresh carrot diet to allow host larvae to develop at ambient conditions. The number of recovered puparia were kept under the same conditions to allow parasitoids and flies to emerge. Thereafter, the parasitoids and flies of each species were counted and uneclosed puparia were dissected to reveal pharate adults which were then handled in the same way as above. 

### 2.3. Data Analysis

Percentages of the parasitoids *D. longicaudata* and *P. cosyrae* landing, probing and ovipositing on the oviposition unit over time (when offered hosts *B. dorsalis and C. cosyra* separately or mixed) were arcsine transformed and then subjected to a repeated measure analysis of variance. A t-test was used to compare landing, probing and oviposition events between the two parasitoids on each host combination. To test the effect of the host combination and parasitoids release sequence on the parasitism rates, percentage of the parasitism for each parasitoid, which is expressed as the total number of parasitoids emerged divided by the total number of exposed larvae, was arcsine transformed and then subjected to analysis of variance. Once significant difference was detected, data were subjected to post-hoc analysis using Tukey test at α = 0.05. Furthermore, host mortality for each parasitoid release sequence was corrected using the mortalities in the control by applying Abbott’s formula (Abbott 1925). Afterwards, data were subjected to a binomial regression in order to test the effect on parasitoids release sequence on the host mortality rate. All analyses were performed using R software version 3.1.1 [73].

## 3. Results

### 3.1. Host Acceptability

**Figure 1:**
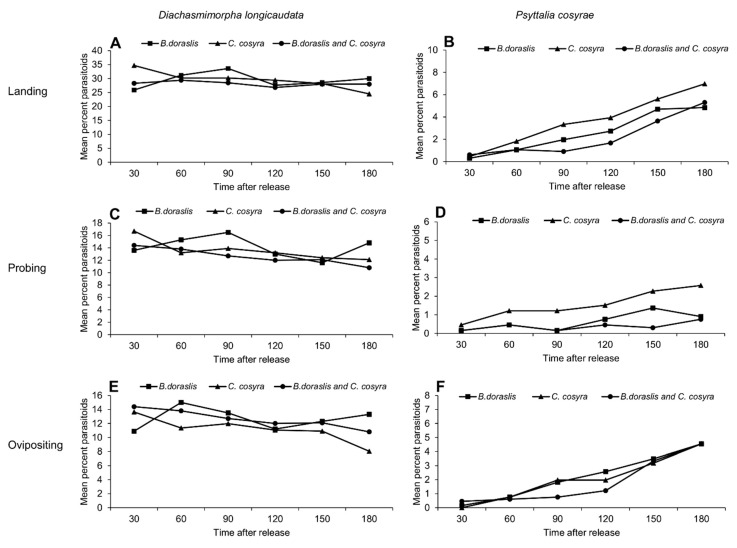
Activity trend of (**A**) *Diachasmimorpha longicaudata* and (**B**) *Psyttalia cosyrae* landing on the oviposition unit; (**C**) *Diachasmimorpha longicaudata* and (**D**) *Psyttalia cosyrae* probing on the oviposition unit; and (**E**) *Diachasmimorpha longicaudata* and (**F**) *Psyttalia cosyra* ovipositing on the oviposition unit over time at 30-min time intervals.

*Diachasmimorpha longicaudata* wasps were faster than *Psyttalia cosyrae* in landing, probing and initiating oviposition in all host combinations tested (Figure 1A–F). In addition, the landing of *D. longicaudata* increased over time and reached a peak at 90 min after release on *B. dorsalis* (*F_5,192_* = 2.489, *p* < 0.05). However, on *C. cosyra*, the landing was higher in the first 30 min, then decreased with an increase in time (*F_5,192_* = 3.691, *p <* 0.01) (Figure 1A). On the other hand, *Psyttalia cosyrae* needed more time to search for the host and its landing significantly increased overtime on all host combinations with a peak at 180 min (*B. dorsalis*; *F_5,192_* = 11.05, *p <* 0.001; *C. cosyra*, *F_5,192_* = 10.61, *p <* 0.001; the combination of both hosts *F_5,192_* = 13.09, *p <* 0.001) (Figure 1B). The probing event was constant for *D. longicaudata* regardless of the host combination (Figure 1C). However, it was significantly higher for *P. cosyrae* on *B. dorsalis* at 150 min (*F_5,192_* = 2.541, *p <* 0.05) and *C. cosyra* at 180 min (*F_5,192_* = 2.914, *p <* 0.05) (Figure 1D). *D. longicaudata* initiated the oviposition in the first 30 min after release on all hosts and the oviposition trend remained constant over time on all hosts combinations except on *B. dorsalis* where the percentage of wasps ovipositing was significantly higher at 60 min after release (*F_5,192_* = 2.174, *p <* 0.05) (Figure 1E). Nonetheless, *P. cosyrae* initiated oviposition at around 60 min after release and the percentage of wasps ovipositing increased gradually at a steady slow pace in all host combinations evaluated and reached the peak at 180 min (*B. dorsalis*, *F_5,192_* = 10.73, *p <* 0.001; *C. cosyra*, *F_5,192_* = 9.128, *p <* 0.001; the combination of both hosts *F_5,192_* = 13.03, *p <* 0.001) (Figure 1F).

**Figure 2:**
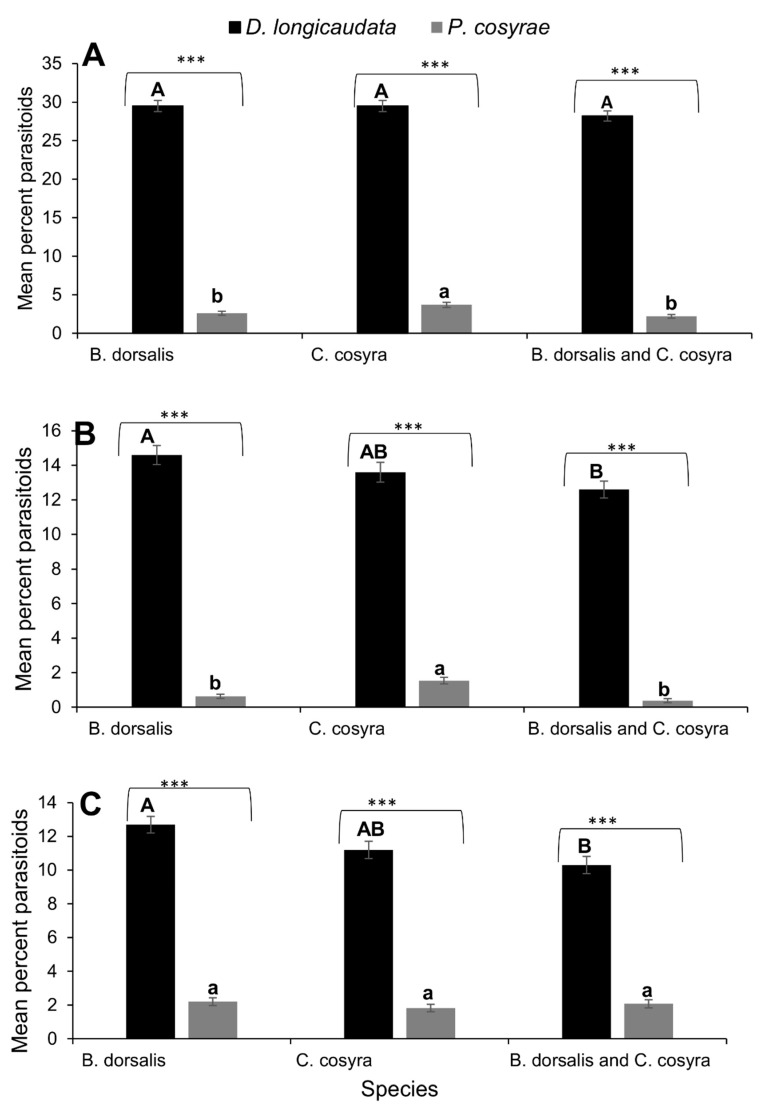
Mean percent (±SE) of parasitoids *Diachasmimorpha longicaudata* and *Psyttalia cosyrae* activities with (**A**) searching, (**B**) probing and (**C**) ovipositing over a period of 180 min on *Bactrocera dorsalis* and *Ceratitis cosyra* as hosts. For each parameter, means superscripted by the same capital or small letters do not differ significantly (repeated measure ANOVA at α = 0.05). *** = *p* < 0.0001 for *t*-test.

For the entire foraging period (180 min), searching events for *D. longicaudata* were comparable regardless of whether the host was solely *B. dorsalis*, *C. cosyra,* or a mixture of the two (*F_2,591_* = 1.182, *p* = 0.30) (Figure 2A). However, it was significantly higher for *P. cosyrae* when *C. cosyra* was the sole host compared to *B. dorsalis* and a mixture of the two hosts (*F_2,591_* = 7.54, *p <* 0.001) (Figure 2A). The comparison between the two parasitoids showed that searching events for *D. longicaudata* were higher than those for *P. cosyrae* in the three host combinations (sole *B. dorsalis*: t = −35.04, *df* = 394, *p <* 0.0001; sole *C. cosyra*: *t* = 33.14, *df* = 394, *p <* 0.0001; mixture of the two hosts: *t* = 36.74, *df* = 394, *p <* 0.0001) (Figure 2A). The probing event for *D. longicaudata* was significantly lower when *B. dorsalis* and *C. cosyra* were mixed, compared to a scenario when the hosts were offered separately (*F_2,591_* = 3.314, *p <* 0.05) (Figure 2B). However, for *P. cosyrae*, probing events were significantly lower on sole *B. dorsalis* and a mixture of both hosts, compared to sole *C. cosyra* (*F_2,591_* = 17.49, *p <* 0.001) (Figure 2B). Additionally, it was significantly higher for *D. longicaudata* on all hosts combinations, compared to *P. cosyrae* (sole *B. dorsalis* t = −24.606, *df* = 394, *p <* 0.0001; sole *C. cosyra*: *t* = 20.081, *df* = 393, *p <* 0.0001; mixture of the two hosts: *t* = 24.334, *df* = 394, *p <* 0.0001) (Figure 2B). The ovipositing events for *D. longicaudata* followed a similar trend to that of probing events, with a lower percentage of oviposition on the mixture of *B. dorsalis* and *C. cosyra* (*F_2,591_* = 5.857, *p <* 0.01) (Figure 2C). However, it was comparable for *P. cosyrae* regardless of whether the host was solely *B. dorsalis*, *C. cosyra*, or a mixture of the two (*F_2,591_* = 0.778, *p =* 0.46). The ovipositing events for *D. longicaudata* were significantly higher compared to those of *P. cosyrae* regardless of host combination (solely *B. dorsalis*: *t* = −19.341, *df* = 394, *p <* 0.0001; solely *C. cosyra*: *t* = 16.145, *df* = 394, *P* <0.0001; mixture of the two hosts: *t* = 15.062, *df* = 394, *p* <0.0001) (Figure 2C).

### 3.2. Effect of Host Combinations and Parasitoid Release Sequence on the Parasitism Rates 

The percent of parasitism was higher for *D. longicaudata* compared to *P. cosyrae* on either *B. dorsalis* or *C. cosyra* irrespective of parasitoid–host combination or sequence of encounter (solely, simultaneously or sequentially) (Table 2). When *D*. *longicaudata* was solely released on different host combinations (*B. dorsalis*, *C. cosyra* or the mixture on both), the parasitism was higher on *C. cosyra* with 78.09%, compared to *B. dorsalis* and the mixture on both (*F*_2,96_ = 23.42, *p <* 0.0001) (Table 2). Similarly, the parasitism of *P. cosyrae* was higher on *C. cosyra* when it was solely released, followed by the mixture on both hosts (*F*_2,96_ = 181.3, *p <* 0.0001). In addition, the parasitism of *P. cosyrae* on *B. dorsalis* was zero, indicating the unsuitability of *B. dorsalis* as a host for this parasitoid. In a simultaneous release, the parasitism outcome for *D*. *longicaudata* was the same on all host combinations (*F*_2,96_ = 1.63, *p =* 0.201), but it was significantly higher for *P. cosyrae* on the mixture of both hosts (*F*_2,96_ = 319.7, *p <* 0.0001) (Table 2). When *D. longicaudata* was released first in sequential releases with *P. cosyrae*, its parasitism was significantly lower on the combination of both hosts, compared to sole *B. dorsalis* or *C. cosyra* (*F*_2,96_ = 14.21, *p <* 0.0001). Nevertheless, it was significantly higher for *P. cosyrae* when both hosts were mixed, compared to sole *C. cosyra* (*F*_2,96_ = 14.39, *p <* 0.0001) (Table 2). A similar trend was observed when *P. cosyrae* was introduced first in sequential releases (*D. longicaudata F*_2,96_ = 18.59, *p <* 0.0001; *P. cosyrae F*_2,96_ = 25.54, *p <* 0.0001) (Table 2).

Parasitoid release sequences on the same host (sole, simultaneous and sequential releases) also had a significant effect on the host parasitism (Table 2). On *B. dorsalis* as a sole host, parasitism of *D. longicaudata* was similar in all release sequences, except in a sequential release where *P. cosyrae* was introduced first (*F*_3,128_ = 2.721, *p <* 0.05) (Table 2). However, on *C. cosyra* as a sole host, parasitism of *D. longicaudata* was significantly higher in a sole release, compared to simultaneous or both scenarios of sequential release (*F*_3,128_ = 24, *p <* 0.0001). When *D. longicaudata* was offered a mixture of *B. dorsalis* and *C. cosyra*, the parasitism outcome was higher in simultaneous release with a parasitism rate of 66.24%, followed by sole release (*F*_3,128_ = 13.1, *p <* 0.0001). For *P. cosyrae* release on *C. cosyra* as a sole host, the parasitism was very low in simultaneous and sequential releases where *D. longicaudata* was introduced first. These parasitism outcomes were 66 times lower, compared to a scenario where *P. cosyrae* was solely released on *C. cosyra* (*F*_3,128_ = 169.8, *p <* 0.0001) (Table 2). However, on a mixture of *B. dorsalis* and *C. cosyra*, the parasitism of *P. cosyrae* was higher in sole release followed by simultaneous release (*F*_3,128_ = 137.2, *p <* 0.0001) (Table 2). 

### 3.3. Effect of Parasitoids Release Combination on the Host Mortality Rate

The mortality rate of the hosts was significantly affected by the parasitoid release sequence (single, simultaneous, or sequential releases) (Table 3). On *B. dorsalis* as a sole host, the mortality rates were comparable in all parasitoids release sequences, except when *D. longicaudata* was released first in sequentially (χ^2^ = 133.34, *df* = 128, *p <* 0.05) (Table 3). Indeed, *P. cosyrae* did not induce mortality on *B. dorsalis*. However, on *C. cosyra* as sole host, the mortality was higher when *D. longicaudata* was solely released (86.3%), which was three times higher compared to the sole release of *P. cosyrae* (χ^2^ = 171.63, *df* = 160, *p <* 0.0001). When *B. dorsalis* and *C. cosyra* were mixed in ratio 50:50, the mortality of both hosts was higher when the two parasitoids were simultaneously released on the hosts (*B. dorsalis*, χ^2^ = 140.84, *df* = 127, *p <* 0.0001; *C. cosyra* χ^2^ = 173.46, *df* = 158, *p <* 0.0001), compared to other release sequences (Table 3)

## 4. Discussion

The present study reports relatively high parasitism of both the invasive *B. dorsalis* and the indigenous *C. cosyra* by the imported and introduced parasitoid *D. longicaudata.* These are further data to support findings by Mohamed et al. [31], who reported that *D. longicaudata* had formed a new association with *C. cosyra.* In our view, in addition to suppressing *B. dorsalis*, this association and high reported parasitism, enhances the management of *C. cosyrae* whose co-evolved parasitoid *P. cosyrae,* is known to cause poor parasitism in its host. Copeland et al. [74], reported a relatively low percent of parasitism of native fruit fly species such as *C. cosyra*, *Ceratitis fasciventris* (Bezzi), (Walker), *Ceratitis rosa* Karsch and *Ceratitis anonae* (Graham) by indigenous parasitoids for example *Psyttalia* sp in Kenya. Hence the new association of *D. longicaudata* with *C. cosyrae* provides a promising efficient natural enemy for the pest. However, under certain competitive interactions, the resulting encounters negatively affect *P. cosyrae* populations especially when the native parasitoid fails to differentiate hosts. Various studies focusing on success of biological control have reported the occurrence of interspecific competition among released natural enemies, which have been extrapolated to explain possible scenarios in open field conditions [75].

*Diachasmimorpha longicaudata* out-performed *P. cosyrae* in all possible parasitoid–host encounters, a situation which could be detrimental to the perpetuation of the indigenous parasitoid when hosts are in limited supply. This is further compounded by the fact that *D. longicaudata* is a better competitor than its native counterpart, as shown by its efficiency in searching for a host, probing and ovipositing. These are quality attributes for an efficient natural enemy and conveys a superior advantage over its competitor. Effective biological control relies on parasitoids that are highly efficient in foraging for a host to minimize time and energy expended in such exercise [57,61]. According to the optimal oviposition theory, female parasitoids should be able to choose hosts that confer a great amount of fitness to the offspring [76]. *Psyttalia cosyrae–B. dorsalis* encounters produced no offspring at all, yet the parasitoid was observed ovipositing in *B. dorsalis* larvae. This could result in reproductive sinks in which failure by *P. cosyrae* to discriminate between viable and unviable hosts could result in wasted reproductive effort and ultimately effect population density. The authors of this study have independently observed *D. longicaudata* frequenting rotten mangoes in orchards especially with poor sanitation. These observations concur with Purcell [77], who observed a similar behavior on rotting guavas infested with *B. dorsalis*. Normally, such fruits contain late second or third instar larvae of fruit flies. Thus, based on knowledge that *P. cosyrae* has a shorter ovipositor and prefers first instar and early second instars, compared to *D. longicaudata* which has a relatively long ovipositor and prefers late second instar larvae [31], we assume, that host density will play a pivotal role in mediating coexistence in field conditions where hosts occur in abundance and choices are wide. This notion is in line with Taylor [67] who reported that host equilibrium has a positive effect on stabilizing parasitoid–host interactions. In the context of intrinsic competition and competitor-free space reported by Paranhos et al. [78], it is possible for *P. cosyrae* to exploit unoccupied niches through specializing in early instar larvae as alluded to above. The authors reported gradual replacement of *Doryctobracon areolatus* (Szepligeti) by *D. longicaudata* in circumstances where free uncontested space was unavailable but recounted coexistence of the two in complex biotic and abiotic environmental conditions where niche separation was unavoidable. This is highly possible considering the diverse availability of domesticated and wild fruits in Sub-Saharan Africa as well as their availability nearly all year round [79]. This scenario presents conducive conditions for avoidance of spatial and temporal interactions thus preventing direct encounters that would otherwise be detrimental to the weaker interspecific competitor such as *P. cosyrae.*

*Psyttalia cosyrae* existence will depend on niche differentiation under field conditions. Similar findings were reported by Pekas et al. [80] in two parasitoids utilizing the same host where the poor competitor realigned its foraging strategy and preference in terms of host size and habitat. Furthermore, coexistence in Opiinae has been shown to occur through resource partitioning and various foraging behaviors resulting in divergence of niches [81]. For example, García-Medel et al. [82] reported that *Doryctobracon crawfordi* (Viereck) preferred foraging on infested fruits fallen to the ground compared to the majority of other neotropical Opiinae parasitizing a wide range of fruit flies. *Psyttalia cosyrae* has managed to maintain its populations in Africa even at low *C. cosyra* densities [74] and thus might be able to utilize narrow pest population niches where the much more active *D. longicaudata* does not occur. Such a scenario has been observed in Mexico where *Doryctobracon areolatus* and *Opius hirtus* (Fischer), parasitoids of *Anastrepha* spp., have perfected their foraging behavior in locating hosts at extremely low densities, and are able to sustain their populations where most parasitoids are unable to do so [82]. It is also possible for niche separation to occur as a result of fruit size [78], considering that *P. cosyrae* has a shorter ovipositor compared to that of *D. longicaudata.*

We further note that spatial separation between the native and the introduced parasitoid is possible, especially considering the reports by Ekesi et al. [83] that the preferred host of *P. cosyrae* (i.e., *C. cosyra*) was slowly being displaced from mango agro-ecological systems by the invasive *B. dorsalis* through exploitative and interference competition. This is a clear case of agro-systems playing an integral role in shaping population and community ecology [79]. In this case, detrimental effects of interspecific competition exerted by the superior competitor can be averted considering that in as much as our results show that *D. longicaudata* is a better competitor than *P. cosyrae,* we report no incidences of massive interference which could potentially affect population dynamics of the native parasitoid. This is consistent with findings of Santos et al. [84], who reported no inhibitory, aggressive or exploitative interspecific competition between *D. longicaudata* and the native parasitoids *Opius bellus, Asobara anastrephae*, *Doryctobracon areolatus* and *Utetes anastrephae* (Braconidae) as well as *Aganaspis pelleranoi* (Figitidae) in Brazil. In our case, separation of the two hosts *B. dorsalis and C. cosyra* in space may eventually limit and restrict the occurrence of *P. cosyrae* in agro-systems dominated by their coevolved host *(C. cosyra).* An interesting scenario was reported in Hawaii in which *F. arisanus* and *Diachasmimorpha tryoni* (Cameron) shared the same niche in controlling *Ceratitis capitata* (Wiedemann). Intense extrinsic and intrinsic competition forced the weaker competitor *D. tryoni* to flee competition and attack non target fruit flies following the establishment of *F. arisanus.*

Our findings also indicated that either parasitoid species was able to parasitize host larvae in the same oviposition unit regardless of which species encountered the host first. In addition, the two parasitoid species could simultaneously encounter hosts at the same time and still manage to parasitize without any interspecific interference. This is encouraging especially with regard to coexistence and combined suppression of the shared host (*C. cosyra*), otherwise lethal interspecific interference competition would have been detrimental to either of the two and their immediate community structure. It is not clear what the outcomes of intrinsic competition are, especially in *C. cosyra* where encapsulation did not occur. According to Cusumano et al. [85], parasitoid interactions require a deep prognosis of both extrinsic and intrinsic competition in order to understand the intricate details shaping populations. In solitary parasitoids, as in our case, elimination of the weaker competitor occurs due to contest competition in larvae or even at egg stage if chemical signals are involved [86].

Previous studies had indicated that *D. longicaudata* was performing poorly on its co-evolved host (*B. dorsalis*) and fears were that the new association with *C. cosyra* could result in abandonment of its host in preference of *C. cosyra* (the parasitoid has been released in Kenya, Tanzania, Mozambique, Benin, Ethiopia, and efforts are currently underway to release in Malawi, Zambia and Zimbabwe). Thus, there were calls to explore for more larval parasitoids to effectively suppress the invasive pest. The good news is that the current study reports a five-fold improvement in *B. dorsalis* parasitism by *D. longicaudata* compared to initial parasitism rates reported by Mohamed et al. [31] which is a good yardstick for the suppression and sustained management of *B. dorsalis* and and *C. cosyra*, though still *D. longicaudata* parasitism on *C. cosyra* is 14% better compared on *B. dorsalis.* The issue of parasitoid interactions remains a hugely understudied area, but recent insights into the intricate relationships leading to coexistence and sharing of hosts in open field conditions continue to be plausible facts shaping parasitoid–parasitoid and parasitoid–host interactions.

## 5. Conclusions

In conclusion, the introduction of *D. longicaudata* in Africa is a welcome move as it potentially benefits the management of both *B. dorsalis* and *C. cosyra* simultaneously. Our findings present a worst-case scenario in which the native parasitoid may be driven into extinction due to the reproductive sink effect and competitive displacement. The best-case scenario will be the possibility for co-existence where the hosts are not limited, and choices are wide open, as evolutionary adjustments occur over time. 

## Figures and Tables

**Table 1:** *Diachasmimorpha longicaudata* and *Psyttalia cosyrae* release strategies, sequences, and densities on two fruit fly hosts *Bactrocera dorsalis* and *Ceratitis cosyra* under laboratory conditions.

Parasitoid Release Sequences	Host Combination	Description of Parasitoid Release Sequences on the Host Combination
*D. longicaudata* alone	*B. dorsalis*	20 *D. longicaudata* on 100 *B. dorsalis* larvae
*P. cosyrae* alone		20 *P. cosyrae* on 100 *B. dorsalis* larvae
*D. longicaudata* alone	*C. cosyra*	20 *D. longicaudata* on 100 *C. cosyra* larvae
*P. cosyrae* alone		20 *P. cosyrae* on 100 *C. cosyra* larvae
*D. longicaudata* alone	*B. dorsalis* and *C. cosyra*	20 *D. longicaudata* on 50 *B. dorsalis* + 50 *C. cosyra* larvae
*P. cosyrae* alone		20 *P. cosyrae* on 50 *B. dorsalis* + 50 *C. cosyra* larvae
*D. longicaudata* and *P. cosyrae*	*B. dorsalis*	10 *D. Longicaudata* + 10 *P. cosyrae* on 100 *B. dorsalis* larvae
*D. longicaudata* and *P. cosyrae*	*C. cosyra*	10 *D. longicaudata* + 10 *P. cosyrae* on 100 *C. cosyra* larvae
*D. longicaudata* and *P. cosyrae*	*B. dorsalis* and *C. cosyra*	10 *D. longicaudata* + 10 *P. cosyrae* on 50 *B. dorsalis* + 50 *C. cosyra* larvae
*D. longicaudata* first, *P. cosyrae* second	*B. dorsalis*	10 *D. longicaudata* first for 3 hrs, followed by 10 *P. cosyrae* for 3 hrs on 100 larvae
*P. cosyrae* first, *D. longicaudata* second		10 *P. cosyrae* first for 3 hrs, followed by 10 *D. longicaudata* for 3 hrs on 100 larvae
*D. longicaudata* first, *P. cosyrae* second	*C. cosyra*	10 *D. longicaudata* first for 3 hrs, followed by 10 *P. cosyrae* for 3 hrs on 100 larvae
*P. cosyrae* first, *D. longicaudata* second		10 *P. cosyrae* first for 3 hrs, followed by 10 *D. longicaudata* for 3 hrs on 100 larvae
*D. longicaudata* first, *P. cosyrae* second	*B. dorsalis* and *C. cosyra*	10 *D. longicaudata*, followed by 10 *P. cosyrae* on 50 *B. dorsalis* + 50 *C. cosyra* larvae
*P. cosyrae* first, *D. longicaudata* second		10 *D. longicaudata*, followed by 10 *P. cosyrae* on 50 *B. dorsalis* + 50 *C. cosyra* larvae

**Table 2:** Mean percent (±SE) of specific parasitism rates (%) of *Diachasmimorpha longicaudata* and *Psyttalia cosyrae* on two fruit fly species *Bactrocera dorsalis* and *Ceratitis cosyra* following various release combinations under laboratory conditions.

Parasitoid Species	Host Species Combination	Parasitoid Releases and Combination on the Host			
		Sole Release	Simultaneous Releases	Sequential Release with *D. longicaudata* First	Sequential Release with *P. cosyrae* First
*D. longicaudata*	Sole *B. dorsalis*	64.33 ± 1.63 bA	60.58 ± 2.46 aAB	62.42 ± 1.41 aAB	57.12 ± 1.47 bB
	Sole *C. cosyra*	78.09 ± 1.43 aA	63.3 ± 2.09 aB	60.21 ± 1.46 aB	65.76 ± 1.28 aB
	*B. dorsalis* and *C. cosyra*	62.42 ± 2.17 bAB	66.24 ± 2.34 aA	49.29 ± 2.12 bC	54.3 ± 1.291 bBC
*P. cosyrae*	Sole *B. dorsalis*	0.00 ± 0.00 b	0.00 ± 0.00 b	0.00 ± 0.00 b	0.00 ± 0.00 b
	Sole *C. cosyra*	20.12 ± 2.07 aA	0.30 ± 0.12 bC	0.24 ± 0.09 bC	2.18 ± 0.395 aB
	*B. dorsalis* and *C. cosyra*	17.47± 1.36 aA	7.06 ± 0.54 aB	1.24 ± 0.28 aC	1.39 ± 0.304 aC

For each parasitoid species, means in rows followed by the same capital letter and in columns followed by the same small letter are not significantly different at *p <* 0.05 (Tukey test).

**Table 3:** Effect of combinations of *Diachasmimorpha longicaudata* and *Psyttalia cosyrae* on two fruit fly species *Bactrocera dorsalis* and *Ceratitis cosyra* mortality rates (mean ± SE%) under laboratory conditions.

Host Species Combination	Parasitoid Releases and Combination on the Host				
	Sole Release of *D. longicaudata*	Sole Release of *P. cosyrae*	Simultaneous Releases	Sequential Release with *D. longicaudata* First	Sequential Release with *P. cosyrae* First
Sole *B. dorsalis*	67.82 ± 2.56 B	-	68.58 ± 4.19 B	77.35 ± 1.64 A	67.97 ± 2.47 B
Sole *C. cosyra*	86.32 ± 1.79 A	26.64 ± 2.99 D	73.27 ± 2.46 C	74.83 ± 2.02 C	80.42 ± 1.99 B
*B. dorsalis* ^§^	70.95 ± 3.48 B	-	80.28 ± 2.70 A	51.06 ± 4.17 D	66.80 ± 3.62 C
*C. cosyra* ^§^	71.18 ± 4.63 B	50.17 ± 3.93 D	79.78 ± 2.55 A	62.25 ± 3.12 C	73.13 ± 3.27 B

^§^*B. dorsalis* and *C. cosyra* are mixed in ratio 1:1. - *P. cosyrae* did not induce mortality on *B. dorsalis*. For each host species, means in the rows followed by same letter are not significantly different at *p <* 0.05 (Tukey test).
